# Conservation Genetics of Threatened *Hippocampus guttulatus* in Vulnerable Habitats in NW Spain: Temporal and Spatial Stability of Wild Populations with Flexible Polygamous Mating System in Captivity

**DOI:** 10.1371/journal.pone.0117538

**Published:** 2015-02-03

**Authors:** Almudena López, Manuel Vera, Miquel Planas, Carmen Bouza

**Affiliations:** 1 Department of Genetics, Faculty of Veterinary Science, Universidade de Santiago de Compostela, Campus de Lugo, Lugo, Spain; 2 Laboratori d’Ictiologia Genètica, Departament of Biology, Faculty of Sciences, Universidad de Girona, Campus de Montilivi, Girona, Spain; 3 Department of Ecology and Marine Resources, Instituto de Investigaciones Marinas (CSIC), Vigo, Spain; The Ohio State University, UNITED STATES

## Abstract

This study was focused on conservation genetics of threatened *Hippocampus guttulatus* on the Atlantic coast of NW Iberian Peninsula. Information about spatial structure and temporal stability of wild populations was obtained based on microsatellite markers, and used for monitoring a captive breeding program firstly initiated in this zone at the facilities of the Institute of Marine Research (Vigo, Spain). No significant major genetic structure was observed regarding the biogeographical barrier of Cape Finisterre. However, two management units under continuous gene flow are proposed based on the allelic differentiation between South-Atlantic and Cantabrian subpopulations, with small to moderate contemporary effective size based on single-sample methods. Temporal stability was observed in South-Atlantic population samples of *H. guttulatus* for the six-year period studied, suggesting large enough effective population size to buffer the effects of genetic drift within the time frame of three generations. Genetic analysis of wild breeders and offspring in captivity since 2009 allowed us to monitor the breeding program founded in 2006 in NW Spain for this species. Similar genetic diversity in the renewed and founder broodstock, regarding the wild population of origin, supports suitable renewal and rearing processes to maintain genetic variation in captivity. Genetic parentage proved single-brood monogamy in the wild and in captivity, but flexible short- and long-term mating system under captive conditions, from strict monogamy to polygamy within and/or among breeding seasons. Family analysis showed high reproductive success in captivity under genetic management assisted by molecular relatedness estimates to avoid inbreeding. This study provides genetic information about *H. guttulatus* in the wild and captivity within an uncovered geographical range for this data deficient species, to be taken into account for management and conservation purposes.

## Introduction

Seahorses (*Hippocampus* spp.) are endangered species due to population decline of many wild populations by means of direct overexploitation, incidental captures, habitat destruction and other anthropogenic perturbations [1–3]. The entire genus *Hippocampus* was listed as threatened by the Convention on International Trade in Endangered Species [4]. The fact that the conservation status of so many seahorse species is Data Deficient [3] demonstrates the challenges in conducting robust conservation assessments [5].

The European long-snouted seahorse, *Hippocampus guttulatus* (Cuvier 1829) is present along the Northeastern Atlantic coast, the Mediterranean and Black Sea. Regionally, it is listed as near threatened, vulnerable or endangered in different European countries [5]. Moreover, it is also included in the list of threatened species and habitats by the Convention for the Protection of the Marine Environment of the North-East Atlantic [6], together with *Cymodocea* meadows and *Zostera* beds, commonly inhabited by seahorses, which reinforces the conservation value of these vulnerable marine ecosystems. Similarly, Spanish legislation has recently included the Mediterranean and Atlantic Iberian populations of *H*. *guttulatus*, *C*. *nodosa* and *Z*. *marina* into the list of wild species under special protection [7].

Despite the huge conservation interest, scarce information is available on population status and trends of European Atlantic seahorses [3]. The major threat to *H*. *guttulatus* is habitat degradation and anthropogenic disturbance, such as destructive fishing practices, coastal development and pollution [3, 6]. On the Galician coast (NW Spain), some commercial fisheries directed at fish, clams or scallops can cause considerable habitat damage, especially to seagrass beds, and it may impact non-targeted species such as seahorses [8]. Field data revealed small census and densities in wild populations of NW Iberian coasts, in agreement with the observation from fishermen and scuba diving associations about the decrease of seahorse populations in the last years [9]. In such context, actions for the development of rearing techniques and conservation of this species have been recently undertaken in NW Spain [9–14].

The primary goal of species conservation is the preservation of viable wild populations in their original habitats. Genetic monitoring is particularly warranted for threatened species that live in vulnerable habitats. The ability to detect genetic differences in time and space for wild populations is critical in species management and conservation of genetic variation [15]. However, investigations of the temporal and spatial genetic structure of marine fish are relatively scarce [16]. Knowledge of seahorse population structure may improve conservation efforts by identifying evolutionary management units and sources of diversity to be used for conservation breeding programs [17]. Previous studies in seahorses based on mitochondrial and microsatellite markers have reported either small genetic differentiation or moderate but significant structure regarding biogeographical discontinuities [17–21]. Geographical structure has also been suggested for Atlantic populations of *H*. *guttulatus* and *H*. *hippocampus*, with barriers to gene flow identified at Cape Finisterre based on mitochondrial DNA [22, 23].

As part of overall conservation strategies, captive breeding programs may be necessary to improve the survival of threatened species that are in vulnerable circumstances [24]. Among the priorities of the conservation breeding are to capture and retain the maximum possible genetic diversity in a limited population and to minimize any deleterious effects, such as artificial selection and disease risks [25]. Genealogical traceability and knowledge about relationships between breeders are crucial data for ensuring successful management of captive stocks, avoiding pairings between closely related individuals [26, 27]. In addition, captive breeding may benefit from incorporating knowledge about the natural breeding system and mate choice, as relevant evolutionary components of population genetic diversity [28]. The knowledge on genetic and social mating systems in seahorses represents essential information for planning conservation mechanisms and strategies in these species [29, 30]. Microsatellite markers have proven to be useful to ascertain the mating system and to investigate parentage and kinship of wild and captive populations of some seahorse species [31–34], including *H*. *guttulatus* [30, 35]. Genetic analysis of different populations of this latter species revealed single maternity per brood [30, 35], and serial monogamy in captivity [30].

This study is included into a multidisciplinary conservation approach on wild populations’ recovery and rearing development of a flagship marine species, the long-snouted seahorse, on the Atlantic Iberian coast (NW Spain). A captive breeding program has been firstly initiated in Europe at the facilities of the Institute of Marine Research (IIM, Vigo, Spain) for research and conservation purposes in this species [9, 10, 13]. In such context, the purpose of the present study was to investigate the spatial and temporal stability of wild populations of this species based on microsatellite markers, and to use such information for the genetic monitoring of the captive broodstock. The specific aims were: i) to assess the genetic structure of wild populations of *H*. *guttulatus* in NW Spain flanking the biogeographical barrier of Cape Finisterre, ii) to evaluate the temporal stability and the effective population size of Atlantic population samples over the six-year period analyzed, iii) to monitor the genetic diversity of the renewed captive broodstock respect to the founder breeders and the wild population of origin, iv) to trace genealogical relationships to evaluate the reproductive success and genetic mating system at short- and long-term (within brood, within season and among seasons) in the conservation program.

## Materials and Methods

### Ethics Statement

All locations were sampled under specific permission by Marine Authorities from the local Government Xunta de Galicia (Consellería de Pesca e Asuntos Marítimos). Non-lethal sampling was carried out from small tissue pieces (dorsal fin or skin filaments) of live specimens for this protected species in the Cantabrian and South Atlantic estuaries under study. Collection, sampling methods, animal maintenance and manipulation practices were conducted in compliance with all bioethics standards of the Spanish Government and approved by the CSIC Bioethics Committee.

### Biological samples and DNA extraction

**Figure 1:**
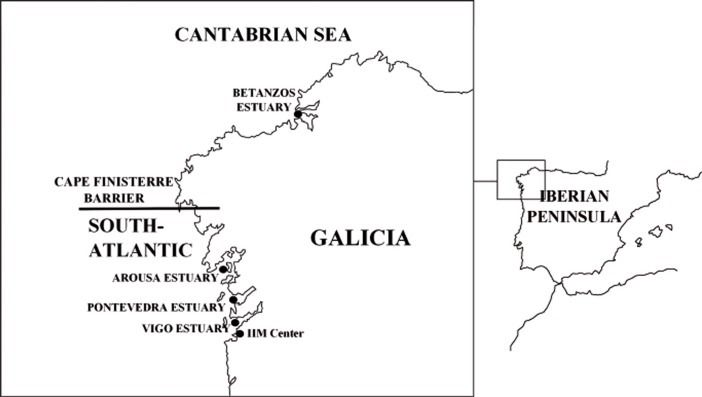
Geographical location of the populations of *Hippocampus guttulatus* analyzed from Galician coasts. The situation of the captive breeding program at the Institute of Marine Research (IIM Center) is also included.

From 2006 until 2012, scuba diving (510 hours) was performed for evaluating wild seahorse resources on Galician coasts (NW Spain). Non-lethal samples of dorsal fin or skin filaments [9] were collected *in situ* from 225 live specimens of *H*. *guttulatus*. They came from Cantabrian (Betanzos 42.1N/8.5W) to South-Atlantic (Arousa 42.3N/8.6W, Pontevedra 42.2N/8.5W, and Vigo 42.1N/8.5W) estuaries, to the north and to the south of Cape Finisterre (Fig. 1). Each estuary comprised 52, 73, 48 and 52 samples, respectively, in the period under study. Temporal samples were available within South-Atlantic estuaries and were distributed in four groups (2006, 2009, 2010 and 2011; Table 1), based on the onset of breeding season of *H*. *guttulatus* in NW Spain (March; [9, 10]).

**Table 1:** Population sampling information of Galician *Hippocampus guttulatus*. Sample size (by sex in brackets; males:females) is indicated for each geographical (see Fig. 1) and temporal sample.

Samplingyear	CS: Cantabrian Sea	SA: South-Atlantic			
	Betanzos	Arousa	Pontevedra	Vigo	Total SA
2006	52 (28:24)	29 (14:15)	2 (1:1)	6 (0:6)	37 (15:22)
2009	nd	11 (7:4)	5 (3:2)	15 (6:9)	31 (16:15)
2010	nd	18 (7:11)	24 (10:14)	2 (2:0)	44 (19:25)
2011	nd	15 (2:13)	17 (11:6)	29 (11:18)	61 (24:37)
All	52 (28:24)	73 (30:43)	48 (25:23)	52 (19:33)	173 (74:99)

Fifty-three out of the wild seahorses sampled were moved to the captive breeding program (Stock09; see Broodstock genetic analysis section) at the Institute of Marine Research (IIM, CSIC, Vigo, Spain; Fig. 1; [9]), under permission of local Government Xunta de Galicia. Since 2009, 814 out of the total 13,948 offspring (6%) obtained from 39 batches of newborn seahorses from the renewed broodstock (mean: 16.8 offspring per batch) were sampled at the IIM facilities for molecular parentage analysis. Among them, 12 and 27 batches were from wild-caught male broods and captive breeding events, respectively. Genomic DNA from non-lethal samples of wild seahorses and from dead young offspring was isolated using NucleoSpin Tissue XS kit (Macherey-Nagel) and Chelex procedure [36], respectively.

### Microsatellite markers

A novel set of 13 microsatellites from those previously reported in the genus *Hippocampus* was selected for population analysis following technical and polymorphism criteria (Table 2; [37–39]). Two multiplex PCR using multiple primer sets were assayed for amplifying and genotyping these microsatellite loci. A first set of six loci useful for parentage analysis in *H*. *guttulatus* was amplified in a single PCR for all wild seahorses and offspring (*Hgu*-USC5, *Hgu*-USC6, *Hgu*-USC7, *Hgu*-USC8, *Hgut*4, *Hgut*6) following López et al. [35]. A second PCR multiplex with the remaining seven loci was amplified for wild seahorses in a volume of 10 μL with 1X Qiagen Multiplex PCR Master Mix, 50 ng of DNA template, and 0.08, 0.10, 0.10, 0.08, 0.15, 0.15, 0.10 μM of each primer for *Hgu*-USC2, *Hgu*-USC4, *Hgu*-USC9, *Hgu*-USC12, *Hgu*-USC13, *Hca*μ25, *Hca*μ36, respectively, using NED, 6-FAM, VIC, VIC, 6-FAM, PET, PET as 5’ fluorescent label for their respective forward primer. PCR conditions consisted of an initial denaturation at 95°C for 15 min, followed by 25 cycles at 94°C for 30 s, 57°C for 90 s and 72°C for 60 s, and a final extension at 60°C for 30 min. PCR products were run on an ABI 3730xl DNA Analyzer (Applied Biosystem), using GeneMapper v4.0 (Applied Biosystems) for genotyping.

**Table 2:** Genetic diversity for 13 microsatellite loci in the Cantabrian (CS06) and South-Atlantic (SA06, SA09, SA10, SA11) wild population samples, and in the renewed captive broodstock (2009–2012) of ***Hippocampus guttulatus***.

Locus	Allelic range^b^	CS06				SA06				SA09				SA10				SA11				Stock09		
		A	AR	H_e_		A	AR	H_e_		A	AR	H_e_		A	AR	H_e_		A	AR	H_e_		A	AR	H_e_
*Hgu*-USC2	141–157	5	4.5	0.522*		4	3.6	0.530		2	2.0	0.506		4	3.7	0.530		5	4.0	0.541		4	3.4	0.516
*Hgu*-USC4	122–146	6	5.3	0.478		3	3.0	0.453		4	4.0	0.450		7	5.4	0.391		6	4.6	0.532		6	4.5	0.434
*Hgu*-USC5^a^	241–277	10	8.8	0.811		8	7.6	0.801		7	7.0	0.768		8	7.3	0.777		11	9.4	0.823		8	7.6	0.779
*Hgu*-USC6^a^	281–328	8	7.3	0.605		8	7.4	0.623		13	13.0	0.841		12	9.9	0.688		14	11.1	0.682		16	12.3	0.730
*Hgu*-USC7^a^	373–413	19	15.9	0.896		14	13.4	0.896		13	12.9	0.905		18	16.2	0.924		19	15.9	0.903		19	16.8	0.915
*Hgu*-USC8^a^	149–187	15	12.9	0.864		12	11.7	0.897		8	8.0	0.837		12	11.2	0.839		13	11.4	0.870		13	12.2	0.864
*Hgu*-USC9	300–330	5	4.5	0.289		6	5.6	0.404		6	6.0	0.303		5	4.8	0.306		6	4.9	0.300		6	5.6	0.360
*Hgu*-USC12	141–153	3	2.8	0.148		3	3.0	0.265		2	2.0	0.097		3	2.7	0.109		3	3.0	0.277		3	2.9	0.176
*Hgu*-USC13	329–333	3	2.9	0.348		3	2.9	0.364		3	3.0	0.396		2	2.0	0.379		3	2.9	0.352		3	2.9	0.343
*Hgut*4^a^	128–220	29	25.3	0.962		30	28.0	0.968		32	31.4	0.968		33	29.2	0.972		35	28.1	0.965		35	28.4	0.968
*Hgut*6^a^	190–358	30	24.9	0.961		24	22.5	0.951		22	21.8	0.938		25	22.5	0.952		29	24.2	0.960		28	24.0	0.959
*Hca*μ25	119–127	5	4.6	0.304		4	3.9	0.426		3	3.0	0.422		4	3.9	0.369		5	4.5	0.369		4	4.0	0.411
*Hca*μ36	188–194	3	3.0	0.533		4	3.8	0.534		2	2.0	0.429		3	2.7	0.518		3	2.7	0.490		3	2.6	0.506
Mean		10.8	9.4	0.594		9.5	8.9	0.624		9.0	8.9	0.605		10.5	9.3	0.597		11.7	9.7	0.620		11.4	9.8	0.612
SD		9.6	8.0	0.280		8.6	8.0	0.248		9.1	8.9	0.283		9.6	8.5	0.281		10.3	8.4	0.260		10.4	8.6	0.268

^a^ Panel of six polymorphic loci useful for parentage analysis [34]

^b^ Allelic range over samples in base pairs.

*A*: Number of alleles; *AR*: Allelic richness based on rarefaction algorithm; *H*_*e*_: Unbiased expected heterozygosity.

*P<0.05: Significant deviation from Hardy-Weinberg expectations after sequential Bonferroni correction.

### Data analysis

Genetic diversity and demography

Allele frequencies were obtained from genotype data (S1 Table) using Fstat v2.9.3.2 [40]. Conformance to Hardy-Weinberg (HW) expectations and linkage disequilibrium were checked using exact tests implemented in Genepop v4.0 [41], applying Bonferroni correction for multiple tests. The presence of null alleles and scoring errors (allele dropout, stuttering) was investigated using Micro-Checker v2.2.3 [42]. Allelic richness (*AR*) was obtained using Fstat. Allele number per locus (*A*), and observed and expected heterozygosity (*H*_*o*_, *H*_*e*_) were estimated using Cervus v3.0 [43].

Contemporary effective population size (*N*_*e*_) was computed using single point and temporal methods [44]. i) *N*_*e*_ in single spatial samples caught in the same year from Cantabrian (CS06) and South-Atlantic (SA06) estuaries was estimated using ONeSAMP v1.2 [45, 46]. *N*_*e*_ prior intervals of 2 to 500 were applied based on the small and unequal population density observed across locations during underwater surveys [9], and assuming that sampling size (37–52) could represent around 10% of the population size [44]. ii) Starting from the most distant temporal samples from South-Atlantic estuaries (SA06 vs. SA11; Table 1), a temporal moment-based estimate of *N*_*e*_ was obtained using NeEstimator v1.3 [47], assuming a generation time of 1.8 years [48].

Signatures of genetic bottlenecks were investigated using the M-value method implemented in M_P_Val [49]. Mean *M*-values over loci and their significance (10,000 replicates) were calculated assuming a proportion of 0.10 for multi-step mutations with mean size of 3.5. A wide range of possible scenarios was covered (*θ* = 4*N*_*e*_ *μ*), from small (*θ* = 0.1) to large (*θ* = 10) pre-bottleneck *N*_*e*_ in natural populations, and also a long-term *N*_*e*_ estimate for each sample derived from *H*_*e*_ (*N*_*e*_ = [(1/(1-*H*_*e*_))^2^–1]/(8*μ*); *μ*: mutation rate [35]) [50, 51], assuming equilibrium and stepwise mutation model (SMM) [49, 52, 53].

Population structure

Allelic differentiation *A*_*ST*_ between pairs of population samples was estimated using Metapop v2.0.a1 [54]. The statistics Jost’s [55] *D*_*EST*_ and the standardized Hedrick’s [56] *G”*_*ST*_ were estimated using GenAlEx v6.5 [57] (999 permutations, 999 bootstraps), since they are not influenced by intrapopulation diversity. *G”*_*ST*_ is further corrected for bias when number of populations is small [56]. Analysis of molecular variance (AMOVA; [58]) was performed using Arlequin v3.11 [59] to assess the distribution of the genetic variation among and within estuaries, and between temporal samples under *a priori* grouping of samples according to their estuarine location (Betanzos, Arousa, Pontevedra and Vigo; see Table 1 and Fig. 1).

Isolation-by-distance over the studied distribution area was evaluated using the correlation of Rousset’s distance measure [60] based on *G”*_*ST*_ against the logarithm of the geographical distances among the four estuaries of origin. Mantel test (30,000 replicates) was conducted to assess the relationship between genetic and geographical distances, using the software IBDWS [61].

Number of population units (*K*) was inferred using the Bayesian MCMC approach implemented in Structure v2.3.1 [62]. Analysis of the whole sampling data was carried out under the admixture ancestral model with correlated allele frequencies [63], without prior population information and also using prior population information since it could aid to detect cryptic structure under small genetic differentiation [64] (burn-in: 50,000; MCMC: 200,000). Ten independent runs were conducted for the *K*s tested (1 to 13) and the mean of Ln probabilities of data (Ln Pr(X|*K*)) across runs were calculated. Posterior probability of each *K* was computed from Bayes’ Rule [62]. The most likely value of *K* was also estimated according to the Evanno’s Δ*K* statistic using Structure Harvester v0.6.94 [65, 66]. Finally, the Structure output for the best Δ*K* was summarized to correct the variance across runs using Clumpp v1.1.2 [67], and graphically displayed using Distruct v1.1 [68].

Despite the departure from HW expectations at one locus and the possible presence of null alleles at very low frequency in another one (see Results), all population structure analyses were based on all loci. The relative coefficients of genetic and allelic differentiation together with the Structure analyses were recalculated excluding these loci and rendered very similar results (see below; S2 Table; S1 Fig.).

Broodstock genetic analysis

The conditions for the maintenance of the individuals in captivity were reported by Planas et al. [9, 12]. Briefly, adult seahorses were kept under temperature (15°C in winter to 19°C in summer) and natural photoperiod (16L:8D in June-July; 10L:14D in December-January) regimes and fed *ad libitum* twice daily on enriched adult *Artemia* (EG, Inve, Spain), supplemented with captured Mysidacea (*Leptomysis* sp. and *Siriella* sp.).

Genetic diversity in the renewed broodstock since 2009 (Stock09) was compared with the Galician wild population based on the complete set of 13 loci, and with the stock founded in 2006 (Stock06; [9]) using the six most polymorphic loci [35]. Departure from HW expectations and linkage disequilibrium were checked using exact tests implemented in Genepop, applying Bonferroni correction for multiple tests. Genetic diversity estimators (*A*, *A*_*R*_, *H*_*e*_), together with theoretical probabilities for exclusion (*Excl*1 and *Excl*2, when the other parent is unknown and known, respectively) and for sibling identity (*SI*) were estimated using Cervus. *D*_*EST*_ and *G”*_*ST*_ between Stock09 and South-Atlantic wild population samples, as well as between Stock09 and Stock06 were estimated using GenAlEx (999 permutations, 999 bootstraps).

Relatedness (*r*) between all pairs of breeders in the renewed Stock09 was computed using Wang estimator [69] (SPAGeDi v1.2; [70]). The midpoints between the expected *r*-values for unrelated (UR; *r* = 0), half-sibs (HS; *r* = 0.25) and full-sibs (FS; *r* = 0.5) kinships were used as thresholds to classify individuals (UR≤0.125<HS<0.375≤FS; [35, 71]).

Parentage analysis of wild and captive progeny

The renewed broodstock (28 M-males and 25 F-females) was distributed in five aquaria (A to E) under unbiased sex-ratio treatment (5–7 M:5–7 F) to improve reproductive success based on previous data [9, 35]. Temporal changes in some aquaria were recorded, either towards male-biased (4–7 M:3–5 F) or female-biased (2–4 M:5–6 F) sex-ratios (Table 3), which were associated with new incorporations from wild populations and eliminations by death or disease. The distribution of breeders would provide 22 possible mating pairs under strict monogamy assumption. Twelve wild-caught males carrying young inside their brood pouch when collected were kept separately until newborn seahorses were released (wild batches); three of these males were sampled in the vicinity of a female during the field survey. Twenty-seven batches of newborn seahorses were obtained from reproductive events in captivity (captive batches). Parentage assignment for all offspring genotyped (S1 Table) in each aquarium was assessed using the exclusion-based method (FAP v3.6; [72]), including all sampled females for parentage analysis of batches from wild-pregnant males. When necessary, maternal genotypes were reconstructed from progeny and paternal arrays using gerud 2.0 [73], and the resulting inferred maternal genotypes were compared with each other and with the population genotype collection for adult females using Cervus.

**Table 3:** Parentage analysis of 39 batches of newborn ***Hippocampus guttulatus*** obtained from wild and captive reproductive events.

Batch	Origin ^a^	Sex-ratio ^b^	Date	N ^c^	Parent (♂-♀)Assignment	*r* ^d^	Full-sibs ^e^	Half-sibssire/dam ^f^	
B1	Captive/A	5♂/5♀	Jul-10	30	G87-G68	-0.083	FS1		
B2	Captive/D	4♂/6♀	Jul-10	30	G81-G83	0.225	FS2		
B7	Captive/D	4♂/6♀	Sep-10	30	G81-G83				
B15	Captive/D	4♂/6♀	Nov-10	29	G81-G83				
B3	Captive/D	2♂/6♀	Aug-10	30	G79-G91	-0.039	FS3		
B4	Wild-Pontevedra	1♂/1♀	Aug-10	2	G98-G97	-0.109	FS4		dHS1
B9	Captive/C	7♂/4♀	Oct-10	30	G98-G97				
B5	Wild-Arousa	1♂/un	Aug-10	27	G104-G159	0.124	FS5		
B6	Wild-Vigo	1♂/un	Aug-10	30	G109-F1	0.312	FS6		
B8	Wild-Arousa	1♂/un	Sep-10	24	G105-G75	0.190	FS7		
B10	Captive/C	7♂/4♀	Oct-10	30	G112-G115	0.083	FS8	sHS1	dHS2
B18	Captive/C	6♂/4♀	May-11	10	G112-G115				
B11	Wild-Betanzos	1♂/un	Oct-10	29	G114-F2	-0.144	FS9	sHS2	
B12	Wild-Betanzos	1♂/un	Oct-10	31	G118-F3	0.240	FS10	sHS3	
B13	Wild-Pontevedra	1♂/un	Oct-10	30	G111-F4	0.002	FS11		
B14	Captive/C	7♂/4♀	Nov-10	30	G113-G97	0.151	FS12	sHS4	dHS1
B16	Captive/C	6♂/3♀	Jan-11	11	G114-G116	-0.090	FS13	sHS2	dHS3
B24	Captive/C	7♂/4♀	Jul-11	19	G114-G116				
B19	Captive/C	6♂/4♀	May-11	20	G118-G116	0.243	FS14	sHS3	dHS3
B21	Wild-Pontevedra	1♂/1♀	Jul-11	1	G169-G168	0.217	FS15		
B22	Wild-Pontevedra	1♂/un	Jul-11	20	G167-F5	0.120	FS16		
B25	Captive/C	7♂/4♀	Sep-11	20	G113-G115	0.315	FS17	sHS4	dHS2
B29	Captive/C	5♂/3♀	Oct-11	20	G113-G115				
B35	Captive/C	4♂/5♀	Jun-12	19	G113-G115				
B26	Captive/D	4♂/4♀	Oct-11	20	G73-G71	-0.086	FS18	sHS5	
B27	Captive/C	6♂/4♀	Oct-11	18	G112-G116	0.196	FS19	sHS1	dHS3
B28	Wild-Vigo	1♂/1♀	Oct-11	19	G171-G176	0.133	FS20	sHS6	dHS4
B30	Captive/B	5♂/5♀	Nov-11	20	G70-G89	0.050	FS21		
B31	Captive/D	4♂/4♀	Nov-11	18	G73-G78	-0.014	FS22	sHS5	
B32	Captive/C	4♂/5♀	Dec-11	12	G177-G172	-0.133	FS23		dHS5
B34	Captive/C	4♂/5♀	Jun-12	19	G171-G172	-0.106	FS24	sHS6	dHS5
B39	Wild-Pontevedra	1♂/un	Jul-12	19	G222-F6	-0.073	FS25		
B41	Wild-Pontevedra	1♂/un	Jul-12	4	G224-G76	-0.113	FS26		
B42	Captive/C	4♂/4♀	Aug-12	16	G214-G178	-0.073	FS27	sHS7	dHS6
B43	Captive/C	4♂/4♀	Sep-12	14	G171-G178	0.006	FS28	sHS6	dHS6
B48	Captive/C	4♂/4♀	Dec-12	9	G171-G178				
B45	Captive/C	4♂/4♀	Sep-12	20	G214-G176	0.251	FS29	sHS7	dHS4
B49	Captive/C	4♂/4♀	Dec-12	4	G214-G176				
B46	Captive/E	4♂/4♀	Oct-12	19	G210-G116	-0.157	FS30		dHS3

^a^ Wild or captive origin

^b^ Observed sex-ratios within aquarium in captivity and for wild-caught males in the wild, either with one known (1♀) or unknown (un) female in the field surveys

^c^ N: Genotyped offspring samples

^d^ *r*: Relatedness coefficient

^e^ FS: Full-sib families

^f^ HS: Half-sibs from sire (s) and dam (d) parents.

## Results

### Genetic diversity of wild population samples

Spatial variation on either side of Cape Finisterre discontinuity

Samples of *H*. *guttulatus* collected in 2006 from Cantabrian Sea (CS06) and South-Atlantic coasts (SA06; Table 1; Fig. 1) were analyzed for spatial comparison northward and southward of the Cape Finisterre, respectively. A single locus showed significant deviation from HW equilibrium after Bonferroni correction (heterozygote excess at *Hgu*-USC2 in CS06). It was not associated to genotyping errors using Micro-Checker, which only showed signs of null alleles at *Hgut*6 at low frequency (0.03) in CS06. There was no evidence of linkage disequilibrium between all pairs of loci tested. There was no indication of spatial change in genetic diversity between CS06 and SA06, with no significant differences in number of alleles, allelic richness and expected heterozygosity (Table 2; *P* = 0.056, *P* = 0.327 and *P* = 0.084, respectively; Wilcoxon tests). The spatial samples showed *M*-ratios around 0.7 which is a diagnostic value of genetic bottlenecks [49] (*M* = 0.679 and 0.697 in CS06 and SA06, respectively). The *M*-ratio obtained in the whole dataset (*M* = 0.737 in CS06+SA06 under continuous gene flow; see below) was also lower than the cutoff value of 0.82 reported for stable natural populations [49, 53]. Most *M*-values reached statistical significance or were marginally significant (under very large pre-bottleneck *N*_*e*_; S3 Table), suggesting that they were not strongly affected by the choice of prior *N*_*e*_ scenario, and, thus, that they represent a reliable evidence of genetic bottleneck [52]. Point estimates of contemporary effective population size (*N*_*e*_) based on single spatial samples were 862 in CS06 (95% CL: 474, 2307) and 200 in SA06 (95% CL: 136, 430).

Small and non-significantly different from zero *D*_*EST*_ (0.003; 95% CI: -0.003, 0.011) and standardized *G”*_*ST*_ (0.005; 95% CI: -0.006, 0.016) values were obtained between CS06 and SA06. Allelic differentiation between both population samples was significantly different from zero at 0.05 level (*A*_*ST*_ = 0.124; 95% CI: 0.020, 0.182), with 26 private alleles although mostly rare at the most variable loci (only three with a frequency above 0.05). Similar results were observed when the only locus deviating from HW (*Hgu-*USC2) was excluded (S2 Table). Heterogeneity of allelic frequencies per locus was only significant at *Hgut*4 (*P* = 0.0003) with two alternative alleles in CS06 (*158; freq. 0.09) and SA06 (*164; freq. 0.07).

Spatial and temporal variation along northwestern Iberian coasts

Results from Structure analyses for the global dataset (all geographical and temporal samples; Table 1) revealed one population unit as the most probable number of clusters (*P*_(*K* = 1)_ = 1; *P*_(*K* = 2–13)_≈0). These same posterior probabilities of *K* were observed in the spatial analyses from the same year (CS06 and SA06). The highest Δ*K* was observed for *K* = 6 and *K* = 2 in the global and spatial analyses, respectively, using Structure Harvester, but none of them was consistent with a temporal or spatial clustering. Thus, the proportion of the sample assigned to each cluster was roughly symmetric (1/*K* in each population) and most individuals were fairly admixed (S1 Fig.), indicative signs of absence of population structure [62]. This could be due to limitations of Δ*K*, which cannot find the best *K* if *K* = 1 [65]. Similar results were observed when *Hgu-*USC2 (with HW deviations) and *Hgut*6 (signs of null alleles at low frequency) were excluded from the global and spatial analysis (highest *P*-value for *K* = 1; best Δ*K* for *K* = 2 and *K* = 3, respectively, with clear signs of genetic homogeneity; S1 Fig.). AMOVA results allocated practically all genetic variation (99.80%) to differences within samples (Variance component = 3.49363; *P* = 0.100). Only 0.47% was assigned to differences among temporal replicates within estuary (Variance component = 0.01651; *P* = 0.112) and no differences were found among estuaries (-0.27% of genetic variation; Variance component = -0.00937; *P* = 0.885). Correlation between genetic and geographical distances among estuaries was not significant (Mantel test; r = 0.297; *P* = 0.294). These data support geographical and temporal genetic stability (i.e. one panmictic unit) of *H*. *guttulatus* in the area under study.

Accordingly, South-Atlantic estuarine samples were grouped in four temporal samples for population monitoring along six-year period (SA06–SA09–SA10–SA11; Table 2). No signs of genotyping errors but of null alleles at *Hgu*-USC7 in SA09 and *Hgu*-USC5 in SA11 (frequency: 0.09 and 0.07, respectively) were found using Micro-Checker. No significant deviations from HW expectations were detected after Bonferroni correction within each of the four temporal samples. Concordance to HW was observed when they were pooled. There were no indications of linkage disequilibrium between all pair of loci tested within temporal samples. No significant differences in genetic diversity were observed for allelic richness and heterozygosity estimators among the four temporal SA samples (Table 2; Wilcoxon tests; *P*>0.05). Neither significant differences in genetic diversity by sex nor heterogeneity of allele frequencies between sexes within temporal samples have been detected. The temporal moment-based estimate of *N*_*e*_ was 1,602 (95% CI: 210, ∞) in the South-Atlantic population sample of *H*. *guttulatus* (SA06 vs. SA11).

According to the global structure analysis, a single population cluster including all temporal samples was also the most probable alternative using the Bayesian analysis (*P*≈1; *K* = 2–4, *P*≈0). The highest Δ*K* was observed for *K* = 2 using Structure Harvester, but with signs of genetic homogeneity (symmetric sample proportion 1/*K* assigned to each cluster and all individuals fairly admixed; S1 Fig.). Small and non-significantly different from zero estimates of genetic differentiation among South-Atlantic temporal samples were observed, both global (*D*_*EST*_: 0.004, 95% CI: -0.002, 0.014; *G”*_*ST*_: 0.006, 95% CI: -0.004, 0.019) and between pairs of samples (*D*_*EST*_ and *G”*_*ST*_ ranges: from -0.004 to 0.009 and from -0.007 to 0.015, respectively; minimum for SA06-SA11 and maximum for SA09-SA11).

### Genetic diversity of the captive population

No significant deviations from HW expectations and absence of linkage disequilibrium were detected in the renewed broodstock comprising 53 wild *H*. *guttulatus* (Stock09). No significant differences in genetic diversity were observed between the Stock09 (*A*_*R*_: 9.8, *H*_*e*_: 0.612; Table 2) and the wild population samples of origin (SA06–09–10–11; mean *A*_*R*_: 9.6, mean *H*_*e*_: 0.607) (*P*>0.05; Wilcoxon and Kruskal-Wallis tests). Similar genetic diversity was detected in the renewed Stock09 (mean *A*_*R*_: 19.3; mean *H*_*e*_: 0.869; Table 2) and the founder Stock06 based on the six most polymorphic loci (*P*>0.05; Wilcoxon test; [35]). These six microsatellite markers provided a useful tool for parentage analysis based on their high combined exclusion probabilities for a false parent when the other parent was unknown (*Excl*1: 0.9985) or known (*Excl*2: 0.9999), and for sibling identity (*SI*: 0.9988). Small and non-significantly different from zero estimates of genetic differentiation were observed between the renewed Stock09 and the founder Stock06 (*G''*_*ST*_ = -0.001, 95% CI: -0.026, 0.024, *P* = 0.47; *D*_*EST*_ = -0.001, 95% CI: -0.024, 0.022, *P* = 0.471), as well as between the renewed Stock09 and the four temporal South-Atlantic wild population samples (*G”*_*ST*_<0.002; *D*_*EST*_<0.001; *P*>0.05; S4 Table).

Pairwise relatedness coefficients (*r*) among breeders within the renewed broodstock of *H*. *guttulatus* ranged from -0.269 to 0.550 (mean *r*: 0.001). Relatedness estimates were low for most pairwise dyads (83%, *r*≤0.125; 72% of them with *r*<0), and high for only a few comparisons between breeders (0.9%, *r*≥0.375). Two of these latter corresponded to specimens of similar size collected from the same estuary and sampling date, thus suggesting real cases of highly-related individuals, beyond inherent error on individual pairwise *r* estimations [27].

### Parentage of wild and captive progeny

Thirty-nine batches of newborn seahorses were analyzed for parentage; 12 of them from wild origin and the remaining obtained from mating events in captivity (Table 3). Wild batches were observed in all estuarine areas since 2010 (Table 3). Captive batches were evenly distributed along the period studied (8, 11 and 8 in 2010, 2011 and 2012, respectively) and across sex-ratio treatments (10, 7 and 10 from unbiased, female- and male-biased, respectively; Table 3).

Each of the 27 captive batches was unambiguously assigned to one parent pair from the captive broodstock. A single mating pair was also assigned in the 12 wild batches (Table 3): i) six of them confirmed either the candidate female in the field (B4, B21, B28) or female genotypes from the same sampling site (B5, B8, B41); ii) in the others, which could not be assigned to any known female in this study, a unique maternal genotype per brood was inferred (coded as F1 to F6; Table 3).

**Figure 2:**
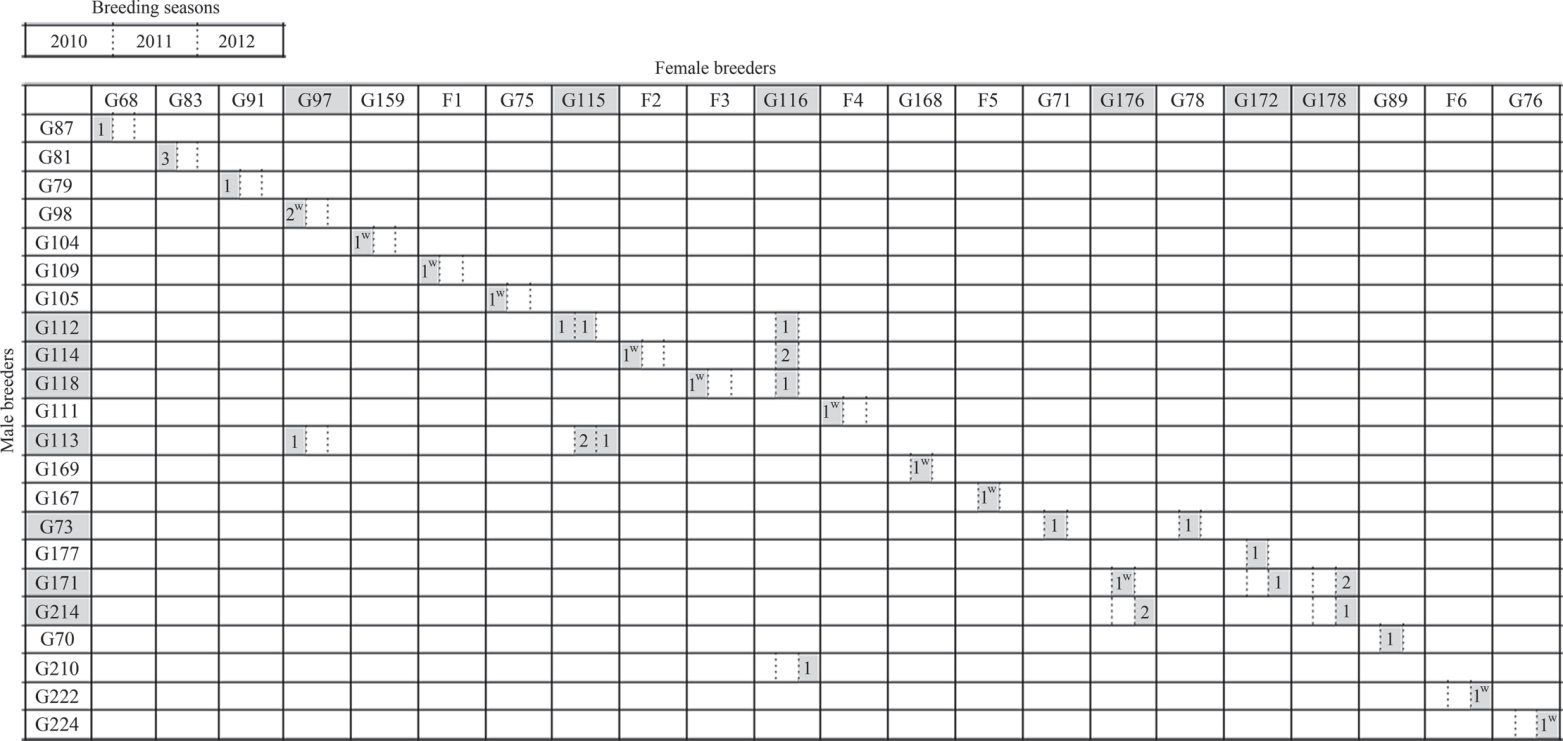
Mating behavior of breeders of *Hippocampus guttulatus* in captivity among three breeding seasons inferred by genetic parentage analysis. First reproductive event of male parents in the wild (^w^), either sampled with one known or unknown female (see Table 3) in the field surveys. Polygynous and polyandrous breeders among breeding seasons were marked in grey.

The parentage analysis of the 39 batches revealed 30 full-sib families (FS), since some captive batches were assigned to the same mating pair (Table 3; Fig. 2). Three captive mating pairs had a single reproductive event along the period under study (16%; FS1, FS3 and FS21), whereas six pairs (31%) were mated monogamously within (FS2, FS13, FS17, FS28 and FS29) and/or among (FS8 and FS17) breeding seasons. Most reproductive units in captivity (53%) showed temporal substitutions of the mating pair within and/or among breeding seasons (6 females and 7 males). This mating behavior determined groups of half-sibs (HS), which shared either sire (7 sHS) or dam (6 dHS) parents (Table 3; Fig. 2). A total of 38 breeders (22 males and 16 females) out of the renewed Stock09, plus six inferred maternal genotypes of wild origin (F1-F6), contributed to all FS and HS families obtained in captivity.

Variable mating behavior was observed from wild to captive conditions (Table 3; Fig. 2). Eight out of 12 male parents of wild batches did not further reproduce under captive conditions (67%). A single male maintained the female partner from the wild (FS4), whereas the others had breeding events in captivity in presence (G171) or not (G114, G118) of the wild-caught female, and even switched the partnership among breeding seasons. Similarly, four known mothers assigned to the wild batches did not further reproduce in captivity (67%); the others showed either monogamous (G97) or polyandrous (G176) mating behavior under captive conditions.

Relatedness between effective mating pairs showed similar ranges in captive (-0.157 for FS30 to 0.315 for FS17) than wild (-0.144 for FS9 to 0.312 for FS6) reproductive events, with lower average in captivity (0.041, SD: 0.152) than in nature (0.075, SD: 0.156). Global relatedness between all pairs of reproductively successful breeders was 0.055 (SD: 0.152), in accordance with the management under low relatedness aquaria and natural mating system.

## Discussion

### Genetic diversity and demography

Average microsatellite diversity over 13 loci in Cantabrian (*A*: 10.8; *H*_*e*_: 0.59) and South-Atlantic (*A*: 9.5; *H*_*e*_: 0.62) population samples of *H*. *guttulatus* in NW Spain was not significantly different to that described over five loci in other European populations of the species [22] (U Mann-Whitney test; *P*>0.05). These values were within the range reported for other seahorse species, such as the endangered *H*. *capensis* (*A*: 10.3; *H*_*e*_: 0.63; [37]) or *H*. *hippocampus* (*A*: 8.0; *H*_*e*_: 0.53; [74]), but lower than reported in marine fish (*A* = 20.6; *H*_*e*_ = 0.79; [75]).

Genetic diversity in *H*. *guttulatus* in this study could be related with population demography, based on the decline reported in some coastal areas of Galicia [9]. In Spain, it seems that *H*. *guttulatus* does not suffer fishing pressure for international trade, and its major threat is related with the vulnerability of its habitat, often degraded by anthropogenic actions, such as coastal development, effect of fishing gear and pollution [3, 6, 76]. Evidence of reduction in population size of the Cantabrian and South-Atlantic contemporary population samples was observed using *M*-ratio tests [49]. Although we did not find signs of severe losses of genetic diversity in the population samples, these signals might be indicative of demographic declines that should not be unnoted [53].

The estimation of contemporary effective population size (*N*_*e*_) can aid to understand the population status of threatened seahorse species, such as *H*. *guttulatus*. Despite the necessity of being cautious of the accuracy of *N*_*e*_ estimation [44], small to moderate contemporary single-sample *N*_*e*_ was observed in South-Atlantic and Cantabrian subpopulations of *H*. *guttulatus*. A larger but less precise *N*_*e*_ estimate was observed in the South-Atlantic subpopulation, with an infinite upper confidence limit. The lower limit that could be an indicator of the lowest possible level of *N*_*e*_ [44] would also alert about small local *N*_*e*_ in such sample. Discrepancy between temporal and single-sample estimates suggests accuracy dependence on the sampling design, and this uncertainty should be taken into account for further management actions [44]. Under continuous gene flow among weakly differentiated Cantabrian and South-Atlantic subpopulations (see below), the *N*_*e*_ estimates would be closer to the metapopulation size of contemporary generations [77]. It could partly explain the higher *N*_*e*_ estimates than local census observed in the field surveys [9]. The low census may be an indication of smaller local population sizes, although certain underestimation due to seahorses’ capacity to camouflage, depth constraints and segregated distributions [18, 78] cannot be ruled out. Regardless of the precise *N*_*e*_ value, cautions about the population persistence on South-Atlantic and Cantabrian coasts should be considered from a conservation perspective in vulnerable habitats, to insure maintenance of genetic variation at the long-term [79].

### Gene flow across the Cape Finisterre barrier

In general, genetic divergence in marine fishes is low due to the high dispersive capabilities through drift of planktonic phases or active adult migration, favored by the lack of geographical barriers [80]. However, several oceanographic features such as ocean current patterns, sea floor topology and thermal gradients, as well as species-specific life-history characteristics may provide some opportunity for separation of populations [23, 81, 82]. The latter may be the case of seahorses, with short juvenile planktonic stage and with low mobility and site fidelity of adults [1, 48], although some evidence suggested occasional long-distance dispersal for some species [1, 23, 83, 84]. Understanding the population structure in seahorses is important for establishing appropriate management units of *H*. *guttulatus* and ensuring best practices for seahorse captive breeding programs.

The Cape Finisterre has been described as a biogeographical boundary associated with the complex Portugal Current System, with different current flows affecting the shores to the north and the south of this cape [85]. This barrier to gene flow has been proposed in different marine species [82, 86], including seahorses *H*. *guttulatus* and *H*. *hippocampus* based on mitochondrial markers [22, 23]. Sufficient gene flow across short to medium distances (tens of km) to homogenize populations has been suggested in seahorses, but across distances >200 km gene flow could be limited [22]. In accordance, in this study analyzing populations of *H*. *guttulatus* from both sides of Cape Finisterre, with a distance about 200 km, no significant genetic structure using microsatellite markers was observed regarding this biogeographical barrier to gene flow (*K* = 1, *P*≈1; *G”*_*ST*_: 0.005, 95% CI: -0.006, 0.016; see also AMOVA and IBD results). Other biogeographical barriers have been suggested eastern (Gulf of Biscay; [87]) or southernmost Cape Finisterre (Estremadura Promontory; [88, 89]). They may be responsible of the differentiation previously detected in *H*. *guttulatus* in absence of samples surrounding Cape Finisterre [22], although discordance among the markers assayed cannot be ruled out. Further mitochondrial and microsatellite analysis in northern Portuguese and eastern Cantabrian populations will aid in defining the management units of *H*. *guttulatus* along the Atlantic Iberian coasts.

This study supports the existence of a single panmictic population of *H*. *guttulatus* throughout Cantabrian and South-Atlantic Galician coasts. However, two management units under continuous gene flow would be proposed based on the allelic distinctiveness (*A*_*ST*_) detected between Cantabrian and South-Atlantic subpopulations. This parameter could be a key point in conservation programs because it gives idea of the potentiality for evolution among subpopulations, since allelic diversity variables are better predictors of long-term adaptation than gene frequency variables [90].

### Temporal stability in South-Atlantic population

Genetic diversity was temporary stable in the South-Atlantic population of *H*. *guttulatus* for the six-year period studied (2006–2012). It suggests that effective population size (*N*_*e*_) could be large enough to buffer the effects of genetic drift, within the time frame of 2–3 generations, although the confidence intervals also alert about the smallest possible *N*_*e*_ in such population (see above). Continuous gene flow could lead homogenization avoiding the loss of genetic variation in the metapopulation of *H*. *guttulatus*. It was associated with low exposure coastal habitats in the underwater surveys, including bays, estuaries and harbors, as other seahorse species [21]. Seahorses in NW Spain were observed on all sediment types, as macroalgae and seagrass, and also on artificial structures [9, 91]. Noteworthy is the stable local population located at the Ribeira harbor in the Arousa Estuary, which reinforces the interest of artificial habitats in shallow waters for conservation of seahorse species [92–94].

### Genetic monitoring of captive breeding

The International Union for Conservation of Nature (IUCN) recognizes the importance of the *ex situ* maintenance for species that are prone to effects of human activities or are likely to become endangered in a very short time [24]. Captive breeding has also made contributions to conservation other than just demographic supplementation, such as research, professional training and public education [79]. Since 2006, a captive breeding program has been established for *H*. *guttulatus* in NW Spain with conservation and research purposes [9]. The genetic diversity of the renewed Stock09 was similar to that of the wild population of origin in this study, and to that of the founder Stock06 [35]. It suggests suitable processes of renewal and maintenance to ensure the genetic representativeness of wild populations of *H*. *guttulatus* in captivity, a key point to guarantee the long-term adaptive potential in conservation programs [95].

The high exclusion and sibling identity probabilities (>0.99) for the most polymorphic loci in the renewed Stock09 allowed the assignment to a single parent pair for all wild and captive batches of newborn seahorses. Each wild batch was compatible with a unique maternal genotype, suggesting different wild female parents (F1 to F6; Table 3; Fig. 2) which would have not been sighted during the field survey. This may point out the existence of cryptic individuals which could partly explain the small local census size observed.

### Within-brood monogamy in the wild and captivity

Interpopulation variation in mating behavior had been reported, including fish [96], and also suggested in seahorses [97]. Parentage analysis in this study proved the single-brood genetic monogamy of wild-caught pregnant males from Galician populations of *H*. *guttulatus* for the first time in the wild. These results are in accordance with those described in Portuguese populations of this species irrespective of the density and sex-ratios [30].

In this study, within-brood monogamy was also observed under captive conditions, confirming previous observations in the species [30, 35]. Captive batches were obtained under female-biased (26%) and unbiased (37%) sex-ratio aquaria, as reported in the founder population [35], but also under male-biased treatment (37%; this study). It suggests that sex ratios do not affect the occurrence of mating events in captivity, as observed for this species in the wild [30].

### Temporal polygamy within and among breeding seasons in captivity

Mate swapping across successive male broods in captivity was observed in the population under study, both within and among breeding seasons. Partner switching was also stated for most wild-caught effective mating pairs (75%) when further reproduced under captive conditions. Mate swapping was observed among different breeding seasons in this study (8%), mostly of females (13%; 5% of males), as reported in this species [30]. However, the previously noted monogamous mating within seasons ([30]; serial monogamy) was not the rule in the population under study. Indeed, more regular mate swapping across broods within a single breeding season (global 21%; 25% of females and 18% of males) was observed (Fig. 2). In seahorses, mate switching within breeding seasons could be harmful for both sexes due to the time cost involved in establishing a new breeding pair [98], being the synchronized cycle with a monogamous mate optimal for reproductive success [30]. Nevertheless, individual’s fecundity also depends on the mating habits, and larger potential fitness benefits can arise from having multiple sexual partners, such as better genes for progeny, higher genetic diversity and improved offspring viability [99]. Our data showed that short and long-term genetic polygamy does exist for socially promiscuous populations of *H*. *guttulatus* in captivity [9]. Geographical variation in male mating behavior has been reported for other syngnathids, related to ecological factors, such as water temperature, adult sex ratio and seagrass biomass [100, 101]. In our study, mate switching was more frequent under male-biased and unbiased (50% and 37.5%, respectively) than female-biased (12.5%) aquaria, suggesting the possible influence of sex ratios on mate switching for the studied population, particularly when there are more males than females.

Naud et al. [102] have pointed out that females in the field may have more chance of reproducing successfully if they mate monogamously, whereas polygynous males could increase their reproductive success. Results in this study did not suggest significant differences in mate switching between males and females under captive conditions, with 32% polygynous males and 38% polyandrous females within the breeders contributing to the offspring (Fig. 2).

The mating systems may have large effects on effective population size (*N*_*e*_). Multiple mating and temporal polygamy can have similar effects on increasing *N*_*e*_ and improving the maintainance of genetic diverstity in a conservation context [103, 104]. The importance of such effects is greatest when population size is small, increasing genetic variation at short term [104]. Also, polygamy could help purge inbreeding depression and decrease extinction rate, compared with a monogamous system in scenarios with small *N*_*e*_ [105]. In this study, a temporal polygamous mating system was observed in *H*. *guttulatus* in captivity, that should be taken into account for management practices with conservation purposes.

### Reproductive success in captivity

Genetic data indicate a flexible mating system for the captive population of *H*. *guttulatus* studied, ranging from strict monogamy to polygamy within and among the three breeding seasons studied (Fig. 2). This could provide an advantage to ensure the reproductive success of the broodstock, allowing the management of breeders under different treatments. Natural mating systems represent an important component of the diversity of populations, recommended to be preserved in captive programs for conservation purposes because of its fundamental influence on breeding success [28]. Molecular monitoring of broodstock management in absence of pedigree data for wild breeders also contributes to preserve genetic diversity and to avoid inbreeding within conservation breeding programs [95]. Low relatedness was observed among most breeders in the renewed Stock09, according to their wild origin. A few dyads with high relatedness estimates (0.9%) could point to pairs of first-degree relatives, which should be held in different aquaria for managing the captive broodstock under minimum inbreeding criteria. Such broodstock organization could explain the lower average kinship observed for mating pairs established in captivity than in the wild (Table 3).

Family data showed higher breeder contribution in the renewed (72%) than in the founder (56%; [35]) broodstock, suggesting an improvement on reproductive success of the captive program under genetic management. This could be also linked to the advances in the phases of nutrition and rearing in captivity [10–14]. The limited effective population size of this captive population based on the reproductively successful breeders (*N*_*e*_ = 37) will maintain short-term genetic diversity (approximately 98% in one generation), but suggests caution about population management in captivity with the aim of ensuring its long-term adaptive potential [79, 95].

## Conclusions

This study adds new genetic information about the conservation status of northwestern Iberian population of *Hippocampus guttulatus* in the wild and under captive conditions, within an uncovered geographical range for this data deficient species [3]. Genetic diversity and demographic inferences on this metapopulation will be useful to be considered in management programs, ideally in combination with evaluation and remediation of underlying causes of associate habitats declines. Supplementation breeding programs to mitigate losses in declining or threatened populations from human activities and/or environmental changes [106, 107] could be considered for further demonstration actions within conservation programs of the long-snouted seahorse in NW Spain. Furthermore, genetic and proactive husbandry research will benefit conservation of wild populations (e.g., methods, life history information [24]) for threatened seahorses that are in vulnerable habitats before they become highly endangered. It will also contribute to stimulate actions on the management of captive populations in Aquarium institutions to be part of wider conservation strategies. The biological and cultural uniqueness of seahorses as flagship species could promote public awareness and change in human behavior regarding shallow coastal waters and marine biodiversity which share the same habitats or are vulnerable to the same threats. The occupancy of artificial habitats could be managed as an opportunity for seahorse conservation accompanied with public education to develop appreciation of the biological richness of coastal ecosystems.

## Supporting Information

S1 Fig Structure plots. Consensus plots across ten Structure’s runs. A) Global analysis based on the set of 13 loci. B) Global analysis excluding *Hgu*-USC2; C) Spatial analysis based on the set of 13 loci. D) Spatial analysis excluding *Hgu*-USC2 and *Hgut*6. E) Temporal analysis based on the set of 13 loci.
(TIF)

S1 Table Genotypes of wild seahorses and offspring analyzed in this study. (XLSX)

S2 Table Pairwise estimates of population differentiation based on the set of 13 loci and excluding *Hgu*-USC2. (XLSX)

S3 Table Bottleneck analyses of *Hippocampus guttulatus* in NW Spain using *M*-ratios. (XLSX)

S4 Table Genetic differentiation of the Stock09 respect to the wild temporal samples from South Atlantic coasts. (XLSX)
