# Supplementary figures and images for: Conservation Genetics of Threatened Hippocampus guttulatus in Vulnerable Habitats in NW Spain: Temporal and Spatial Stability of Wild Populations with Flexible Polygamous Mating System in Captivity
Source: PLoS One. 2015 Feb 3;10(2):e0117538. doi: 10.1371/journal.pone.0117538 (PMC4315495; doi:10.1371/journal.pone.0117538)

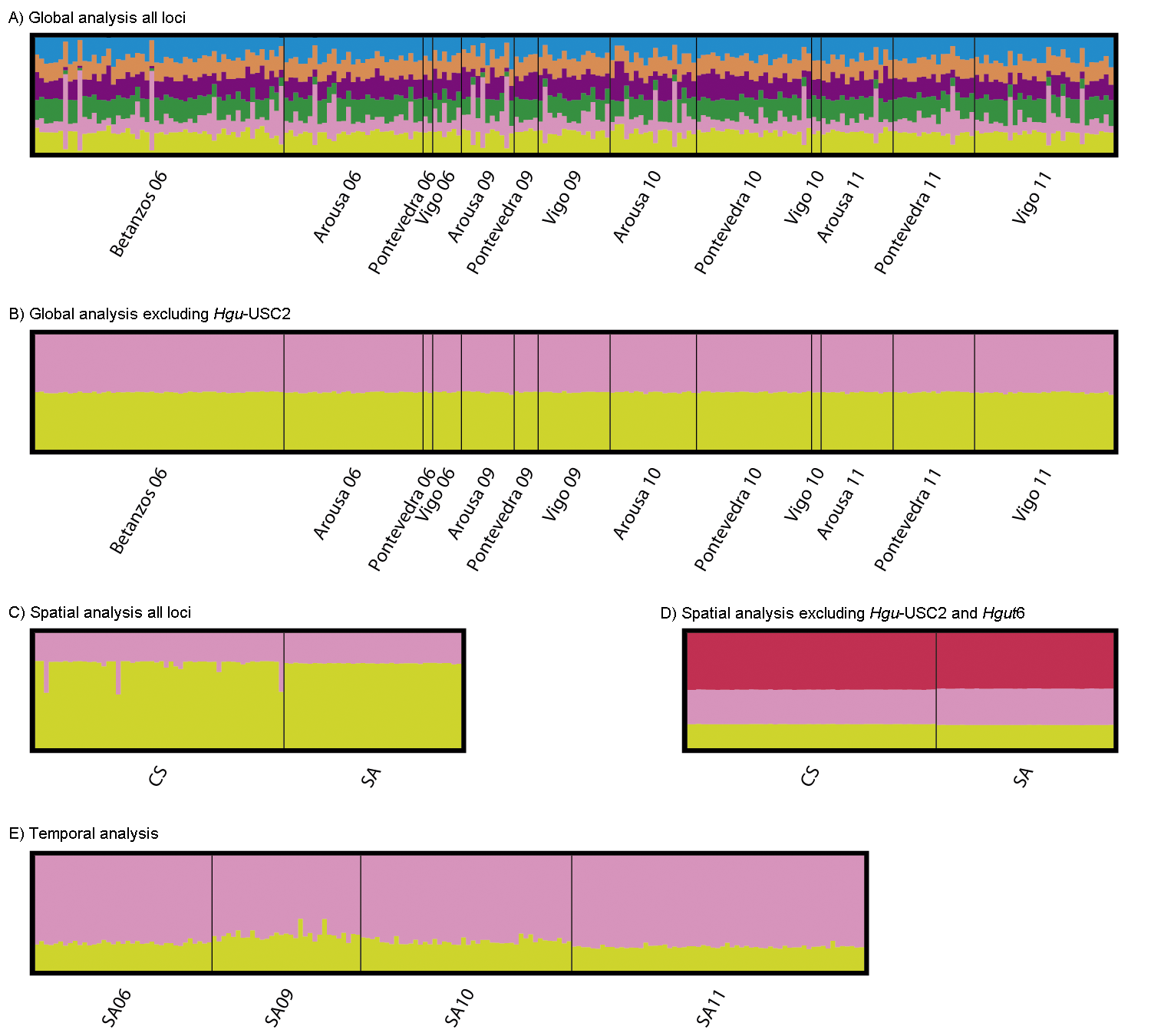

Supplement: S1 Fig — Consensus plots across ten Structure’s runs. A) Global analysis based on the set of 13 loci. B) Global analysis excluding Hgu-USC2; C) Spatial analysis based on the set of 13 loci. D) Spatial analysis excluding Hgu-USC2 and Hgut6. E) Temporal analysis based on the set of 13 loci. (TIF) [file pone.0117538.s001.tif]
